# PReS13-SPK-1579: Biologic agents for the treatment of rheumatic diseases

**DOI:** 10.1186/1546-0096-11-S2-I27

**Published:** 2013-12-05

**Authors:** P Emery

**Affiliations:** 1 University of Leeds, Leeds, UK

## 

Since the middle 1990s biologics have been available for rheumatic diseases. The initial experiments showed efficacy for symptoms and signs but concern was expressed about tachyphylaxis and absence of a true DMARD effect. Combining the antibodies with methotrexate answered some of these criticisms and since that time the number of agents available for rheumatoid arthritis, psoriatic arthritis and spondyloarthropathy has grown.

It is clear these agents work, what is less clear is how they should be used to produce optimum cost benefit. The ever expanding market has attracted a large number of pharmaceutical players to this area and this shows no sign of abating. Also there are number of new drugs including oral synthetic DMARDs becoming available.

## Disclosure of interest

None declared.

